# Soda consumption and risk of dementia: The Northern Manhattan Study

**DOI:** 10.1002/alz.089066

**Published:** 2025-01-09

**Authors:** Arielle Farhi, Tatjana Rundek, Christian Agudelo, Tali Elfassy, Clinton B. Wright, Mitchell S.V. Elkind, Jose Gutierrez, Hannah Gardener

**Affiliations:** ^1^ University of Miami Miller School of Medicine, Miami, FL USA; ^2^ NINDS, Bethesda, MD USA; ^3^ American Heart Association, Dallas, TX USA; ^4^ Columbia University, New York, NY USA

## Abstract

**Background:**

Soda consumption has been associated with vascular risk factors and events. While diet quality is understood to impact dementia risk, little is known about soda consumption in relation to dementia. We hypothesized that regular soda and diet soda consumption were associated with an increased dementia risk.

**Method:**

We utilized data from 959 participants in the Northern Manhattan study, a longitudinal population‐based multi‐ethnic cohort. Regular and diet soda consumption were assessed by food frequency questionnaire at baseline (1993‐2001) and examined continuously (sodas/week) and categorically. Incident dementia was adjudicated during follow‐up after a series of comprehensive neuropsychological and clinical assessments. We used logistic regression models to examine the associations between regular and diet soda consumption and risk of dementia, adjusting for demographics and vascular risk factors (body mass index, diet quality, smoking history, alcohol use, physical activity level).

**Result:**

Of 959 participants with recorded data on soda intake and neuropsychological assessments (mean baseline age 64±8 years, 40% men, 64% Hispanic, 16% non‐Hispanic White, 18% non‐Hispanic Black), 174 (18%) developed dementia over an average of 11 years of follow‐up. 4.9% of participants drank regular soda >1/day and 2.3% of participants drank diet soda >1/day. Consumption of diet soda was associated with an increased risk of dementia (diet soda/week OR = 1.04, 95% CI 1.00‐1.08), adjusting for sociodemographics and vascular risk factors. Those who consumed more than one diet soda per day had a 3.91‐fold increased risk compared to <1/day (95% CI 1.31‐11.63), and this association was especially strong for non‐Hispanic Black participants (OR = 10.01, p<0.05). The association was not apparent after excluding those with obesity or the metabolic syndrome (N = 476, diet soda/week OR = 1.03, 0.94‐1.13). An association between regular soda consumption and incident dementia was only observed after excluding those with obesity or the metabolic syndrome (regular soda/week full cohort: 1.01, 0.98‐1.04; restricted cohort: OR = 1.05, 1.00‐1.10).

**Conclusion:**

The results support a potential increased risk of dementia associated with frequent consumption of regular and diet soda, but further research is needed in diverse study populations, with great attention paid to the confounding and mediating impacts of cardiometabolic risk factors and the potential for effect modification by race.

